# Innate and adaptive immune responses that control lymph-borne viruses in the draining lymph node

**DOI:** 10.1038/s41423-024-01188-0

**Published:** 2024-06-25

**Authors:** Carolina R. Melo-Silva, Luis J. Sigal

**Affiliations:** Department of Microbiology and Immunology, Thomas Jefferson University, Bluemle Life Sciences Building Room 709, 233 South 10th Street, Philadelphia, PA 19107 USA

**Keywords:** Virus infection, lymph borne virus, lymph node, virus control, innate immunity, adaptive immunity, dendritic cells, Langerhans cells, macrophages, inflammatory monocytes, natural killer cells, CD8+ T-cells., Infection, Innate immunity

## Abstract

The interstitial fluids in tissues are constantly drained into the lymph nodes (LNs) as lymph through afferent lymphatic vessels and from LNs into the blood through efferent lymphatics. LNs are strategically positioned and have the appropriate cellular composition to serve as sites of adaptive immune initiation against invading pathogens. However, for lymph-borne viruses, which disseminate from the entry site to other tissues through the lymphatic system, immune cells in the draining LN (dLN) also play critical roles in curbing systemic viral dissemination during primary and secondary infections. Lymph-borne viruses in tissues can be transported to dLNs as free virions in the lymph or within infected cells. Regardless of the entry mechanism, infected myeloid antigen-presenting cells, including various subtypes of dendritic cells, inflammatory monocytes, and macrophages, play a critical role in initiating the innate immune response within the dLN. This innate immune response involves cellular crosstalk between infected and bystander innate immune cells that ultimately produce type I interferons (IFN-Is) and other cytokines and recruit inflammatory monocytes and natural killer (NK) cells. IFN-I and NK cell cytotoxicity can restrict systemic viral spread during primary infections and prevent serious disease. Additionally, the memory CD8^+^ T-cells that reside or rapidly migrate to the dLN can contribute to disease prevention during secondary viral infections. This review explores the intricate innate immune responses orchestrated within dLNs that contain primary viral infections and the role of memory CD8^+^ T-cells following secondary infection or CD8^+^ T-cell vaccination.

## Introduction

The immune system must continuously survey the body to defend against invading pathogens. In vertebrates, much of this immune surveillance occurs in lymph nodes (LNs), a specialized secondary lymphoid organ that constantly receives molecules, cells, and, when present, pathogens from tissues through afferent lymphatic vessels. LNs mostly comprise hematopoietic immune cells, including a majority of adaptive B and T lymphocytes and a relatively small proportion of innate immune cells, such as macrophages, monocytes, dendritic cells (DCs), natural killer (NK) cells, and other innate lymphoid cells. LNs also contain a small but complex population of parenchymal cells that includes high endothelial cells (HECs), lymphatic endothelial cells (LECs), fibroblastic reticular cells (FRCs), and follicular dendritic cells (FDCs) [1]. These parenchymal cells provide structural and essential developmental signals to immune cells and directly contribute to their function [2, 3].

**Fig. 1:**
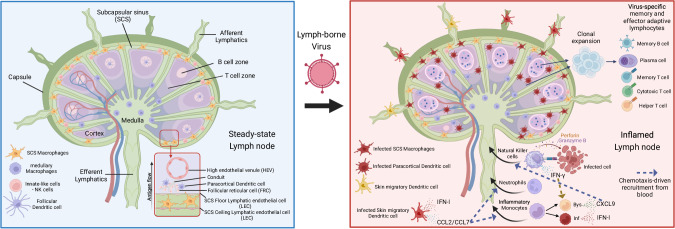
Innate and adaptive immune responses in dLNs induced by lymph-borne viruses. *Left: The lymph node tissue and cellular structure at steady state.* Lymph fluid drained from tissues through afferent lymphatics flows unidirectionally from the SCS to the medullary region and exits through efferent lymphatics. At the SCS and cortical interfaces, a complex web of cells (SCS macrophages, paracortical DCs, LECs, FRCs), conduits, and PLVAP fibrils filter antigens and pathogens into the cortex. Within the cortical B- and T-cell zones, paracortical and follicular DCs activate antigen-specific lymphocytes. *Right: Inflamed dLN.* Upon infection with a lymph-borne virus, infected myeloid APCs (SCS macrophages, paracortical DCs, and skin mDCs) initiate innate immune responses that curb virus dissemination and clonal expansion of adaptive lymphocytes, which are essential for definitive virus control and immune memory. In ECTV-infected animals, infected skin mDCs upregulate Mult-1 and produce IFN-I and CCL2/7, which recruit iMOs from the blood. Mult-1 on mDCs binds NKG2D expressed on LN-resident NK cells, which secrete IFN-γ. IFN-γ signaling on bystander iMOs induces CXCL9 production, which recruits circulating NK cells from the blood. iMOs that become infected stop producing CXCL9 and secrete IFN-I. IFNAR signaling in NK cells induces optimal cytotoxic effector function. The IFN-I transcription of iMOs is increased by *Irf7* and *Cgas* upregulation. NK cell and IFN-I effector functions synergize to restrain virus dissemination from the dLN to target organs (created with Biorender^TM^)

Lymph nodes have a highly organized structure composed of a cortex, a medulla, and a capsule (Fig. 1*, left*). The cortex contains follicles surrounded internally by the medulla and efferent lymphatic vessels. The capsular stromal tissue supports and connects the cortex and medulla with afferent lymphatic vessels, which drain the tissue interstitial fluids. Within each follicle, a B-cell-rich zone is surrounded by a T-cell zone. Between follicles, a cortical interfollicular region houses medullar sinus CD169^+^ F4/80^+^ macrophages, lymphatic sinus DCs, CCL19^lo^ T-zone reticular cells, and high endothelial venules [1, 3–6].

The subcapsular sinus (SCS), located between the capsule and the cortex, is lined by LECs, forming an SCS ceiling and floor. Unbiased transcriptional studies have shown the existence of multiple LEC subsets that populate different parts of the LN and serve key immunological functions [3]. The LECs at the SCS floor constitute a barrier that prevents molecules larger than 70 kilodaltons (kDa) from entering the LN parenchyma. However, LECs also form small diaphragms composed of plasmalemma vesicle-associated protein (PLVAP) [7] that connect the lumen of the SCS with the follicles through a “sponge-like” network of conduits made of FRC and lined by an extracellular matrix produced by the FRCs. Thus, the LECs of the floor serve as sieves that retain large molecules in the SCS while permitting the rapid passage of antigens and molecules smaller than 70 kDa, such as cytokines, from the SCC to the follicles through the conduits [4, 8].

The SCS contains a diverse cell population that includes CD169^+^ F4/80^-^ SCS macrophages, marginal reticular cells, innate-like lymphocytes [9], NK cells, memory CD8^+^ T-cells, follicular memory CD4^+^ T-cells, and memory B-cells. Therefore, the SCS is a site of intense cellular crosstalk, proliferation, and rapid generation of plasma cells from the memory B-cell pool.

SCS macrophages, which have a dendritic morphology, trap pathogens and molecules larger than 70 kDa that enter the lymph node through the afferent lymphatics. SCS macrophages constantly surveil the lymph at a steady state to respond to pathogenic and environmental stimuli and directly interact with B-cells [10].

Thus, LNs possess a complex channeling system composed of the SCS and follicular conduits that can deliver a variety of antigens; pathogens; proinflammatory molecules; and cytokines to different LN compartments, efferent lymphatics, and blood. Here, we review the major immune response events that can occur in the local draining lymph node (dLN) following viral infections to reduce systemic viral dissemination and orchestrate adaptive immunity.

## The pathogenesis of the virus determines the role of the dLN in its control

After entering their host *via* the skin or mucosal surface, many viruses disseminate through a lymphohematogenous route to reach their final target organs. These viruses are known as “lymph-borne”. The routes and means of entry of lymph-borne viruses into their hosts vary. Many poxviruses are lymph-borne. The poxvirus ectromelia virus (ECTV), the ethiological agent of mousepox, is a textbook example of a lymph-borne viruses that enters the body through the skin [11]. ECTV is a natural pathogen of the mouse. It is genetically and morphologically highly related to variola virus (VARV) and Mpox virus (MPOXV), which cause smallpox and Mpox, respectively, in humans, and vaccinia virus (VACV), which serves as the smallpox and Mpox vaccine [11–14]. ECTV naturally enters its murine host through microabrasions of the foot and, experimentally, by footpad injection. Whether naturally or experimentally acquired, ECTV replication in the footpad is substantial and often results in necrosis and loss of the limb, hence the name of the virus [15]. In susceptible mouse strains, ECTV rapidly spreads from the footpad to the dLN and subsequently to the blood and liver. ECTV then replicates massively in hepatocytes, causing acute death, before the mice have time to establish an adaptive immune response. In mousepox-resistant strains, such as C57BL/6 (B6), spread to the liver occurs more slowly. Thus, the mice survive the infection without mousepox symptoms because they have enough time to mount an adaptive immune response. Footpad infection of the mouse with ECTV is one of the best models to study lymph-borne virus pathogenesis. Human lymph-borne viruses can enter the body through different routes and means. MPOXV enters its host through mucosal surfaces and skin abrasions by contact [16, 17], the flaviviruses West Nile virus (WNV) and dengue virus (DENV) penetrate the skin through mosquito bites [18–20], VARV and the Morbillivirus measles virus (MV) invade their hosts within cough droplets *via* the respiratory tract, and the enteroviruses poliovirus (PV) and coxsackie B virus (CVB) use an oral route of infection, most commonly in contaminated water [21–25].

The innate immune response in the dLN during primary lymph-borne virus infections may reduce virus dissemination to target organs [5, 10, 26–34]. As exemplified by ECTV in mousepox-resistant strains, such as B6, this delay in virus spread within the dLN may be crucial to allow enough time for adaptive immune cells to expand and control highly pathogenic viruses. Defects in the innate immune response in the dLN during primary infections, such as those caused by deficiencies in certain NK cell receptors, can lead to susceptibility to lethal viral infection [26, 27]. During secondary viral infections, virus containment by memory CD8^+^ T-cells in the dLN can replace the virus-controlling role of the innate immune response [35]. Thus, memory CD8^+^ T-cells in the dLN can play a crucial role in the early protection of target organs from lymph-borne viruses.

The dLN is vital for initiating the adaptive immune response to non-lymph-borne viruses. However, controlling their spread is unnecessary because the primary target organ is generally infected earlier than the dLN. Thus, for non-lymph-borne viruses, such as influenza A virus (IAV) [36–38], the dLN is required to orchestrate the adaptive immune response, while the restriction of viral replication in the lung depends on the local recruitment and activation of innate immune and memory cells by infected alveolar cells and macrophages. The focus of this review is on lymph-borne viruses.

## Myeloid antigen-presenting cells play a major role in the entry of lymph-borne viruses into the dLN

Viruses are obligatory intracellular pathogens that vary widely in size [39]. After entering their host, some lymph-borne viruses drain through afferent lymphatics to the dLN as free virions. Multiple studies have shown that, a few hours after infection of the footpad, vesicular stomatitis virus (VSV) [10, 34], replication-deficient vaccinia virus (VACV) of the modified Ankara virus (MVA) strain at high doses of 10^8^ plaque-forming units (pfu) [40], and alphaviruses such as Zika virus [19, 41] drain as cell-free virions to the popliteal dLN and enter the SCS, where SCS macrophages can retain viral particles and curb their dissemination [10, 33, 34, 40]. Interestingly, one of these studies showed that SCS macrophages poorly retained virus-sized 200 nm latex beads [10]. This finding suggested that SCS macrophages recognize viral pathogens through specific receptors and not by particle size. More recently, a specialized population of SCS CD11b^+^ DCs was shown to actively scan the lymph with motile dendrites and capture 40–200 nm fluorescent microspheres [42]. Whether this capture was receptor-mediated or promiscuous was not determined. In addition, LECs expressing the scavenger receptor MARCO but not CD169^+^ macrophages were shown to contribute to controlling Chikungunya virus and other alphaviruses in the dLN [43]. Notably, Reynoso et al. showed that wild-type VACV inoculated into the footpad at high doses (10^6^-10^8^ pfu) passed through the SCS and entered dLN conduits as free virions to infect paracortical DCs one hour post-infection [44].

Lymph-borne viruses can also be transported to the dLN within infected cells. The most common virus-transporting cells are of myeloid origin. For example, infected migratory DCs (mDCs) from the skin can shuttle virions from the footpad to the dLNs [30, 33, 45]. Many poxviruses, such as VARV for humans and ECTV for mice, have high species specificity. ECTV can productively infect and spread in mice much more efficiently than VACV. Thus, after small-dose footpad inoculation, ECTV rapidly infects local parenchymal cells and skin-resident mDCs. Following inoculation with only 3×10^3^ pfu in the footpad, ECTV arrived at the dLN 1-2 days post-infection (dpi) in infected mDCs that migrated from the skin. Blocking mDC migration by local inoculation of the footpad with pertussis toxin (PT) [46–48] prevented virus dissemination to the dLN [30]. Notably, in PT-treated mice, the virus loads were reduced in the dLN at two dpi but increased in the spleen at seven dpi. PT treatment of the footpad also increased the lethality of the infection [30]. These results suggest that, after low input of a species-specific poxvirus, virions are initially transported to the dLN within infected mDCs rather than by cell-free lymphatic trafficking. This mDC transport is crucial for mounting an effective antiviral response and is important because natural infections likely involve low virion inputs. It has also been reported that the CD8^+^ T-cell response to herpes simplex virus requires mDC migration from the skin to the dLN [45]. Another study showed that the entry of murine gammaherpesvirus 68 (MHV68) into the dLN was not mediated by Lyz2^+^ cells, such as SCS macrophages, but by CD11c^+^ cells, possibly DCs [33]. More recently, Wang et al. demonstrated that the *Aedes aegypti* mosquito neutrophil recruitment protein, which is secreted in mosquito saliva, indirectly promotes the recruitment of dengue and Zika virus-susceptible myeloid cells to the skin, which can then disseminate these viruses to the dLN [49].

Some viruses can be disseminated to the dLN as free virions and within cells. For example, after a low dose of alphavirus in the footpad, virus trafficking to the dLN occurs both as cell-free virions for up to 4 hours and by infected inflammatory monocytes (iMOs) at 8-18 hours postinfection [41].

Why some viruses disseminate as free virions and others within cells is unclear. The dissemination mode could depend on multiple factors, such as their replication properties, size, infection dose, or the availability of susceptible cells at the entry site.

Whether transported to the dLN within infected cells or as free virions, the cells involved in lymph-borne virus entry and retention in the dLN are generally myeloid cells characterized by their expression of class II major histocompatibility complex (MHC-II) molecules. These cells, generally known as antigen-presenting cells (APCs), include various types of DCs and monocytes/macrophages. In addition, reporter and viral gene expression analysis indicated that most infected cells in the dLN are myeloid APCs and B-cells [30–33, 44, 50–53]. Notably, in the dLN of ECTV-infected mice, myeloid APCs are infected at much greater frequencies than are B-cells [32]. Moreover, at the early infection stages, infected myeloid APCs but not B-cells produce the proinflammatory cytokines and chemokines required for efficient innate and adaptive immunity [50]. Therefore, myeloid APCs are key players in the early protective innate immune response in the dLN.

## The innate immune response in the dLN curtails the systemic dissemination of lymph-borne viruses

Once lymph-borne viruses reach the dLN as free virions or within cells, infected APCs initiate the antiviral response (Fig. 1, *right*). The general features of this response include the upregulation of cytokines and stress-associated molecules and the presentation of antigens to adaptive T-cells. The cytokines and stress-associated molecules produced by infected APCs prime and activate neighboring bystander-uninfected cells for a faster and optimal response to the virus and recruitment of additional innate immune cells from the blood to the dLN. APCs may produce type I interferons (IFN-Is), a family of antiviral cytokines frequently required for efficient immune responses and host survival [25, 54–59]. They may also produce other cytokines required for the recruitment of CD11b^+^Ly6C^hi^ iMOs, CD11b^+^Ly6G^hi^ neutrophils, and NKp46^+^ Eomes^+^ nnatural killer (NK) cells, which further enhance the antiviral immune response by amplifying cytokine production or killing infected cells [60]. The events initiated by infected APCs reduce viral loads within the dLN and virus dissemination to the bloodstream and target organs. The specific APCs and cytokines involved in this process may differ for different viruses, the mechanism of entry to the dLN, and whether the APC is infected or bystander.

When cell-free VSV virions reach the dLN, some of the first APCs they infect are SCS macrophages [10, 33, 40, 61]. The depletion of SCS macrophages with clodronate or diphtheria toxin (DT) abrogates virus retention in the dLN, indicating that SCS macrophages may be important for decreasing viral dissemination. Additionally, intrinsic viral infection activates the inflammasome of SCS macrophages, inducing their release of cytokines and interaction with memory B-cells that migrate from the cortex to the SCS [10, 62]. Following MVA-induced inflammasome activation, infected SCS macrophages die, releasing inflammatory and “danger” signals to other cells in the dLN. Local inflammation then results in the recruitment of iMOs, neutrophils, and NK cells [62, 63].

Skin mDCs, including Langerhans cells (LCs), CD103^+^CD207^+^ type 1 conventional DCs (cDC1s), and CD103^-^CD207^-^ type 2 conventional DCs (cDC2s), carry ECTV from the footpad to the dLN. Of these, only LCs are critical for initiating the innate immune response to ECTV in the dLN [30]. The accumulation of infected and bystander skin-derived mDCs in the dLN begins at one dpi and peaks at two dpi. At three dpi, very few mDCs are still present in the dLN, possibly because they migrate to other organs, die from the infection, or are killed by NK cells. Within the dLN, infected skin-derived mDCs upregulate multiple immune response genes, including the chemokines CCL2 and CCL7, which recruit iMOs from the blood [31, 32]. In addition, skin-derived mDCs induce IFN-γ production by LN-resident NK cells and other type 1 innate lymphoid cells (see below).

Following ECTV infection, the transcriptional profiles of bystander and infected iMOs and B-cells in the dLN are vastly different. Both bystander iMOs and B-cells upregulate antigen presentation and interferon-stimulated genes (ISGs), while infected iMOs and B-cells downregulate antigen presentation genes and poorly upregulate ISGs. Moreover, bystander and infected iMOs, but not B-cells, differentially contribute to the complex cytokine milieu of the infected dLN by upregulating mostly nonoverlapping subsets of cytokine genes [50].

Regardless of the mechanism by which lymph-borne viruses reach the dLN, depletion or defects in the innate immune cells described above compromise or severely reduce the ability of the host to slow virus replication and dissemination to vital organs. Collectively, APCs perform a complex, choreographed innate immune response to combat viruses in the dLN, where different types of infected and bystander APCs play unique roles. As discussed below, the downstream protective mechanisms initiated by infected APCs involve: **1)** IFN-I production in the dLN, **2)** activation and recruitment of NK cells to the dLN, and **3)** activation of primary and secondary adaptive immune responses. However, infected APCs can also contribute to the systemic viral dissemination of lymph-borne viruses.

### IFN-I production in the dLN

IFN-Is are a family of cytokines encoded by intron-less genes that are highly conserved in mammals. Humans and mice encode one IFN-β, more than a dozen IFN-α [64], and other IFN-I subtypes whose functions are poorly understood. All IFN-I subtypes signal through the interferon-alpha receptor (IFNAR), which is composed of the IFNAR1 and IFNAR2 subunits and is ubiquitously expressed in all tissues and cells. IFN-I binding to IFNAR upregulates a large set of ISGs that activate immune and nonimmune cells and directly target different steps of the viral replication cycle [65, 66]. Defects in IFN-I signaling in mice and humans increase their susceptibility to multiple viral infections and the adverse effects of attenuated live-virus vaccines [25, 54–56]. Many viruses encode proteins that specifically inhibit different steps of the IFN-I signaling cascade. These viral proteins play important roles as virulence and host restriction factors for virus replication and pathogenesis [67–71].

Viruses activate pathogen-recognition receptors (PRRs), such as membrane-bound Toll-like receptors, cytosolic RNA-sensing RIG-I and MDA5, or DNA-sensing cGAS. TLRs activate the adaptors MyD88 and TRIFF, RIG-I and MDA5 activate MAVS, and cGAS activates STING through the messenger molecule cGAMP. Downstream of these adapters are the transcription factors NF-κB, IRF3, and IRF7, which can activate the transcription of genes encoding IFN-I and other molecules [18, 72–76]. The genes encoding the IFN-β and IFN-α subtypes are differentially activated because their promoters are composed of different regulatory elements. This differential regulation of IFN-I subtypes is highly conserved among different species [77], suggesting that IFN-I is a critical component of the antiviral response. The human and mouse IFN-β genes can be activated by NF-κB, IRF3, and IRF7 [78–80]. IRF3 and IRF7 regulate mouse IFN-α4 and human IFN-α1 and α13, while all other IFN-α genes are regulated only by IRF7 [81]. Because IRF7 is an ISG, the expression of most IFN-α genes requires previous exposure to IFN-I.

In vitro, most cells can produce IFN-I. However, in vivo, most IFN-I-producing cells are myeloid APCs [30, 32, 82–85]. Among APCs, iMOs, DCs, and plasmacytoid DCs (pDCs) can produce IFN-I in infected animals or humans. After footpad infection of mice with ECTV, infected and uninfected mDCs that migrate to the dLN and the iMOs that are recruited and become infected are the largest populations of IFN-I-producing cells at 1 (mDCs) and 2-3 dpi (iMOs) [29, 30, 32, 50]. IFNAR, cGAS, STING, TLR9, MyD88, NF-κB, and IRF7, but not IRF3, are required to constrain ECTV spread from the dLN to the liver and to survive infection. Notably, the TLR9-MyD88 pathway is required to recruit iMOs to the dLN but not for their transcription of IFN-I. The iMOs that become infected in the dLN use the cGAS/STING/NF-κB pathway to transcribe IFN-β and the cGAS/STING/IRF7 pathway to transcribe IFN-α [57, 68, 84, 86]. Bulk RNA sequencing of sorted infected and bystander iMOs from the dLN of mice infected with ECTV showed that infected iMOs upregulate the transcription of *Nfkb1* (encoding the p105/p50 subunits of NF-κB) [50]. Thus, intrinsic infection may initiate IFN-I transcription through the induction of IFN-β by NF-κB, which is further amplified and expanded to IFN-α by IRF7 in bystander cells as they become infected.

IFN-I production may begin at the entry site, further amplified by cells in the dLN, and may extend to distant organs as the virus spreads [87]. IFN-I produced by APCs acts on surrounding cells to upregulate ISGs and prime them for potential intrinsic infection. Although all cells express IFNAR, not all cells require IFN-I signaling for host resistance to viral infections. Multiple studies using bone marrow chimeras and conditional knockout mice have helped identify which cells must express IFNAR1 so that the host can control systemic viral dissemination [19, 88], prevent viral invasion of the CNS and other specialized organs [88, 89], or survive infection.

To resist mousepox, NK cells and iMOs require intrinsic IFNAR1. In contrast, IFNAR1 is dispensable in adaptive lymphocytes and nonhematopoietic cells, even though the critical targets of ECTV are hepatocytes [29]. In contrast, for ZIKV, the expression of IFNAR1 in bone marrow-derived cells or parenchymal cells reduces viremia and prevents viral entry into the CNS [88]. Additionally, the specific ablation of IFNAR1 in SCS macrophages is insufficient to disrupt the resistance of wild-type mice to ZIKV, but it increases systemic viral dissemination [19]. In the case of herpes simplex virus, LECs in IFNAR1-deficient animals are permissive to infection, leading to edema and loss of LECs in the dLN [90]. Thus, mammals have evolved an antiviral circuit that sounds an efficient alarm for the presence of viral pathogens, which can be read by all cells. The message transmitted restricts cellular tropism for some viruses, curbs virus replication inside the dLN to reduce systemic viral dissemination, and prevents or reduces viral spread to specialized vital organs.

### Activation and recruitment of NK cells to the dLN

NK cells are innate lymphoid cells (ILCs), which, together with type 1 lymphoid cells (ILC1s), are Group 1 ILCs characterized by their production of IFN-γ. NK cells, classical cytotoxic CD8^+^ T-cells (CTLs), and the less recognized cytotoxic CD4^+^ T-cells (CD4^+^ CTLs) have similar cytolytic and IFN-γ-producing functions [91]. However, the activation mechanisms of T-cells and NK cells are very different. T-cells become activated when their antigen-specific T-cell receptor recognizes antigenic peptides presented on major histocompatibility (MHC) molecules. On the other hand, NK cell activation is regulated by the signaling balance of inhibitory and activating receptors. Most of the genes encoding these NK cell receptors cluster in a genomic region known in rodents and humans as the NK gene complex (NKC) [92, 93]. The high susceptibility of some inbred mouse strains to MCMV and ECTV was mapped to the NKC [94, 95]. These findings provide initial evidence indicating that NK cells and their receptors are necessary to resist viral infections. Mutations and allelic variability of NK cell receptors in humans are also associated with severe outcomes of viral infections [96–98]. Mark Buller’s group subsequently confirmed the critical role of NK cells in the control of ECTV [99].

Following ECTV footpad infection, circulating NK cells rapidly migrate from the blood to the dLN. NK cell depletion results in faster spread to the liver, indicating a critical role for NK cells in restricting viral spread from the dLN [26]. Notably, IFN-I produced by infected iMOs in the dLN is necessary for this NK cell restriction of viral dissemination [29]. Interestingly, aging and chronic LCMV infection cause defects in NK cell recruitment to the dLN, resulting in high susceptibility to lethal mousepox [28, 100]. NK cell recruitment from the blood to the dLN or tissues was also observed in mice and nonhuman primates infected with other viruses, such as mouse cytomegalovirus, Murid herpesvirus, Ebola virus, MPXV, and simian immunodeficiency virus [60, 61, 63, 101, 102].

The activating NK cell receptor NKG2D is present in rodents and humans and is important for mice to survive mousepox [26, 27]. Following footpad infection with ECTV, the mDCs that migrate to the dLN upregulate the NKG2D ligand Mult-1 in response to TLR9 and MyD88 signaling to induce IFN-γ production, mostly in LN-resident immature NK cells but also in a few LN-resident ILC1s. This IFN-γ induces the production of the chemokine CXCL9 in bystander iMOs, which recruits mature circulating NK cells from the blood to the dLN. The recruited NK cells further amplify the production of IFN-γ and kill infected cells in the dLN.

CD94 is encoded in the NK gene complex and is essential for the ability of NK cells to control the spread of ECTV from the dLN [27]. CD94 forms a heterodimeric inhibitory receptor with NKG2A or activating heterodimeric receptors with NKG2C and NKG2E [103]. The ligands for CD94-NKG2 heterodimers are the nonclassical MHC-I molecule Qa-1^b^ in mice [104, 105] and HLA-E in humans [106–108]. Experiments in mice have demonstrated that CD94 is essential for restricting the spread of ECTV from the dLN [27]. Within an infected mouse, bystander immune cells upregulate Qa-1^b^ and become protected from NK cell killing, while infected immune cells downregulate Qa-1^b^ to be killed by NK cells [50, 109]. Thus, the inhibitory signal provided by NKG2A/CD94 binding to Qa-1^b^, or HLA-E [110], is critical for NK cells to discriminate between infected cells that must be killed and bystander cells that must be spared.

When recruited to an infected organ, NK cells are exposed to proinflammatory cytokines, which increase the expression of the cytotoxic proteins pore-forming protein (perforin, Prf) and granzyme B (GzmB). The cytokines IL-12, IL-15, IL-18, IL-21, and IFN-I, which are upregulated in the dLNs of infected animals or individuals, enhance NK cell effector functions [32, 50, 60, 82, 111–114]. Notably, conditional deletion of IFNAR1 in NK cells affects NK cell maturation, the expression of Prf and GzmB, and the in vivo killing capacity in ECTV-infected mice [29]. A lack of NK cells [26, 99, 115]; defects in NK cell effector mechanisms, such as deletion of Prf [116], GzmB [117], and IFN-γ [118]; or defects in NK cell activation [26, 27, 96–98, 109, 119, 120] result in high susceptibility to acute lethal viruses, such as ECTV in mice, or an increase in the burden of chronic viruses, such as hepatitis B in humans. Thus, NK cells represent a key innate mechanism restricting lymph-borne virus dissemination from the dLN to other organs [26, 27, 61, 63].

### Activation of primary and secondary adaptive immune responses in the dLN

Successfully mounting an effective innate immune response in the dLN temporarily curtails pathogen spread, providing time to develop antigen-specific T- and B-cell responses that can eliminate the virus and evolve into long-term memory to prevent subsequent infections. In addition to initiating the complex chain of innate immune events that ultimately results in IFN-I and NK cell activation, APCs also present viral antigens on MHC-I and MHC-II molecules to CD8^+^ and CD4^+^ T-cells, respectively, to induce and sustain the expansion of virus-specific T- and B-cells in the dLN. The proliferation of naïve B-cells, their differentiation into virus-specific antibody-producing plasma cells, and the expansion and differentiation of naïve T-cells into B-cell helpers and antiviral cytotoxic T-cell effectors result in short- and long-term immunity and viral clearance. This is mediated by antibody neutralization of viral particles and the killing of infected cells through antibody- and cell-mediated cytotoxicity. The dLN is the main site for the induction of adaptive immune responses to viral pathogens and vaccines in animals [121–123] and humans [124, 125].

Following viral infection, APCs become infected or take up antigens in peripheral tissues or the dLN. These APCs [123, 126] and other innate immune cells, such as NK [127] and NKT [128] cells, secrete a complex array of cytokines that culminate in the activation and recruitment of immune cells to the dLN. The recruited immune populations include NK cells, lymphocytes, iMOs, and neutrophils [129]. These immune activation events lead to the formation of a multicellular follicular structure called the germinal center (GC), where follicular DCs and CD4^+^ T follicular helper cells interact and prime B-cells to divide and differentiate into high-affinity plasma cells and memory cells.

Experiments with fluorescently-labeled molecules and particles showed that B-cells interact with SCS macrophages to acquire antigens and transport immune complexes to follicles in the dLN [10, 130, 131]. However, antibody responses induced by IAV, lymphocytic choriomeningitis virus, and VSV require B-cell priming by DCs [126, 132, 133]. Protective humoral responses also require B-cell stimulation or “help” from follicular CXCR5^+^ CD4 helper T-cells for antibody class switching and affinity maturation [134–137]. CD4^+^ T-cell help is important for long-term B-cell memory and serum antibodies following vaccination and viral infection [138–143]. IAV-driven IFN-I induces the upregulation of the integrin CD11b on B1 B-cells, driving their accumulation in the dLN and their production of low-affinity, virus-specific IgM antibodies that help control the virus until high-affinity IgG and IgA antibody responses develop [144].

The immunological cues necessary for the induction of plasma and memory B-cells in the dLN by lymph-borne viruses must be better understood. Primary infections with lymph-borne viruses provide long-lasting protection from secondary infections [145–150]. However, due to the complexity of mapping polyclonal primary responses in vivo, most studies dissecting events and factors driving memory and effector adaptive responses have focused on non-live vaccines and transgenic animal models with a restricted B-cell repertoire.

Understanding how long-lasting memory and protective responses are induced in the dLN is particularly important for vaccination and natural infections. When infected with lymph-borne WNV, germinal center B-cells differentiate into high-affinity plasma cells and memory cells of variable affinity [151]. Similar results were observed following non-lymph-borne IAV infection or vaccination [152, 153]. Memory B-cells express CD73, CD80, PD-L2, and the transcriptional repressor Bach2 [153, 154]. The biological importance of generating low-affinity memory cells is poorly understood, but it may be associated with cross-protection from similar or emergent pathogens. For instance, low-affinity memory B-cells from WNV-infected mice clonally expand after Japanese encephalitis virus infection and differentiate into cross-reactive B-cell blasts in a germinal center-independent manner [151].

In inflamed dLNs, resident and newly recruited T-cells upregulate CD69 and are prevented from accessing the cortical sinus and exiting the dLN [155]. Naïve and memory antiviral CD8^+^ T-cells are predominantly primed by tissue-resident and dLN-resident DCs [42, 156, 157]. This priming depends on DCs secreting CXCR3-binding chemokines and the expression of CXCR3 by CD8^+^ T-cells [123, 158, 159]. After priming, CD8^+^ T-cells proliferate and differentiate into effector and memory cells. The balance between effector and memory CD8^+^ T-cells depends on the levels of CXCR3- and CCR7-binding chemokines produced by APCs [160].

In the ECTV mouse model, vaccination-induced memory CD8^+^ T-cells protect susceptible mice from lethal mousepox by restricting systemic virus spread from the dLN. This finding provided some of the first lines of evidence demonstrating that the dLN restricts the spread of lymph-borne viruses [35]. Moreover, mice with CD94 NK cell deficiency or depleted of NK cells are protected from lethal mousepox by memory CD8^+^ T-cells induced by immunization with peptide-pulsed bone marrow-derived DCs [161]. This finding suggested that the IFN-γ and cytotoxic effector functions of adaptive memory CD8^+^ T-cells can fully replace the similar spread-limiting effector functions of innate NK cells.

The memory CD8^+^ T-cells that protect against systemic viral spread in the dLN could originate in memory T-cells that recirculate in the blood after primary infection or vaccination. Alternatively, protection could be mediated by LN-resident CD69^+^ tissue-resident memory T-cells (Trms) that migrate to the LN from nonlymphoid tissues. For example, skin Trms migrate to skin-dLNs after secondary antigen exposure, becoming skin-derived LN-Trms that protect against subsequent rounds of viral entry through the skin [162].

## Final remarks

The control and immunosurveillance of lymph-borne viruses highly depend on the innate and adaptive immune responses initiated in the dLN by infected myeloid APCs. While IFN-I and NK cell responses are essential for curbing primary lymph-borne viral infections, control of secondary infections by lymph-borne viruses can be achieved by adaptive memory lymphocytes in the dLN. Complex, highly choreographed crosstalk among bystander and infected innate immune cells occurs in the dLN during the first days following viral infection to induce these protective innate and adaptive immune responses. The outcomes of these innate immune cell interactions are: 1) the recruitment of immune cells from the blood, 2) the production of IFN-I, and 3) the activation of NK cells, in which IFN-I and IFN-γ play pivotal roles.

While some details of the innate immune events necessary for both the recruitment of immune cells from the blood and the activation of NK cells during primary lymph-borne virus infections have been described for a few viruses, the early cellular crosstalk initiated by infected myeloid APCs in the dLN that culminates in the generation of protective long-term immune memory is less well defined. Further investigation of the early immune events induced in the dLN by vaccination or infection with a lymph-borne virus may help elucidate key immunological steps necessary for generating long-term, central, and tissue-resident memory.
